# Mortality Rate Associated with Admission Hyperglycemia in Traumatic Femoral Fracture Patients Is Greater Than Non-Diabetic Normoglycemic Patients but Not Diabetic Normoglycemic Patients

**DOI:** 10.3390/ijerph15010028

**Published:** 2017-12-25

**Authors:** Cheng-Shyuan Rau, Shao-Chun Wu, Yi-Chun Chen, Peng-Chen Chien, Hsiao-Yun Hsieh, Pao-Jen Kuo, Ching-Hua Hsieh

**Affiliations:** 1 Department of Neurosurgery, Kaohsiung Chang Gung Memorial Hospital, Chang Gung University College of Medicine, Kaohsiung 83301, Taiwan; ersh2127@cloud.cgmh.org.tw; 2 Department of Anesthesiology, Kaohsiung Chang Gung Memorial Hospital, Chang Gung University College of Medicine, Kaohsiung 83301, Taiwan; shaochunwu@gmail.com; 3 Department of Plastic Surgery, Kaohsiung Chang Gung Memorial Hospital, Chang Gung University College of Medicine, Kaohsiung 83301, Taiwan; libe320@yahoo.com.tw (Y.-C.C.); VENU_CHIEN@hotmail.com (P.-C.C.); sylvia19870714@hotmail.com (H.-Y.H.); bow110470@gmail.com (P.-J.K.)

**Keywords:** femoral fracture, admission hyperglycemia, stress-induced hyperglycemia, diabetic hyperglycemia, diabetes mellitus, mortality

## Abstract

*Background:* Admission hyperglycemia is associated with increased morbidity and mortality in trauma patients. However, admission hyperglycemia is not only associated with stress-induced hyperglycemia (SIH) but also with diabetic hyperglycemia (DH); furthermore, patients with normoglycemia may not only have non-diabetic normoglycemia (NDN) but also have a possibility of diabetic normoglycemia (DN), with the diabetes under control. This study aimed to assess the effects of SIH and DH on the mortality outcomes of traumatic femoral fracture patients with NDN and DN. *Methods:* Admission hyperglycemia was diagnosed as a serum glucose ≥200 mg/dL upon arrival at the emergency department. Diabetes mellitus (DM) was determined by patient history and/or admission HbA1c ≥ 6.5%. DH and SIH were diagnosed by admission hyperglycemia in patients with and without DM. DN and NDN were determined by absence of admission hyperglycemia in patients with and without DM. These patients were allocated into four groups: SIH (*n* = 75), DH (*n* = 280), DN (*n* = 309), and NDN (*n* = 1326), with detailed information retracted from the Trauma Registry System at a level I trauma center between 1 January 2009, and 31 December 2016. Patients with incomplete registered data were excluded. The adjusted odds ratios (AORs) and 95% confidence intervals (CIs) for mortality were estimated through a stepwise model selection of a multiple regression model that was adjusted by controlling the cofounding variables such age, sex, co-morbidities, and Injury Severity Score. *Results:* Compared to NDN, a 9.8-fold (95% CI 1.54–62.05; *p* = 0.016) and a 5.8-fold (95% CI 1.46–22.67; *p* = 0.012) increase in the adjusted mortality odds ratio of patients with SIH and DH, respectively, were found in this study. In addition, the adjusted odds of mortality between SIH (AOR = 0.3; 95% CI 0.03–2.99; *p* = 0.302) as well as DH patients (AOR = 0.6; 95% CI 0.20–1.89; *p* = 0.394) and DN patients had no significant difference. *Conclusions:* This study demonstrated that SIH and DH patients with traumatic femoral fractures had higher mortality when compared with NDN patients, but not when compared with DN patients, with or without adjustment of the differences in patient’s age, sex, co-morbidities, and injury severity.

## 1. Background

Femoral fractures are the most common consequence of falls in older people associated with an increased risk of morbidity and mortality [1,2]. Acute fractures induce the secretion of stress hormone, resulting in insulin resistance and subsequent hyperglycemia in injured patients [3]. This form of hyperglycemia secondary to a stress response, known as stress-induced hyperglycemia (SIH), commonly occurs in patients with trauma [4,5,6,7,8], burn injuries [9], stroke [10,11], and myocardial infarction [12,13]. The stress response is characterized by excessive gluconeogenesis, glycogenolysis, and insulin resistance and can result in up to 10 times greater adrenal cortical output [14]. The pathophysiology of SIH is thought to reflect relative insulin deficiency and temporary insulin resistance, which was caused by the overwhelming activation of pro-inflammatory mediators (tumor necrosis factor-α, interleukin-6) and counter-regulatory hormone excesses [15]. In such metabolic milieus, the insulin concentrations in the plasma are inadequate to compensate for hyperglycemia [16] and fails to suppress hepatic gluconeogenesis [17]. In trauma patients, SIH is associated with higher morbidity and mortality rates [8,10,18]. The occurrence of acute myocardial infarction in SIH patients with hip fracture was higher than that of the non-hyperglycemia group (12.46% vs. 6.41%, *p* < 0.05), with the highest incidence in the initial three days after a hip fracture [19]. 

In addition, admission hyperglycemia is associated with a high morbidity and mortality in critically ill [4,20] and non-critically ill trauma patients [8]. Among these trauma patients, those with hyperglycemia had a more pronounced mortality than any other type of surgical patients [21]. However, admission hyperglycemia does not only occur in SIH but also in diabetic hyperglycemia (DH), and the patients with normoglycemia may not only have non-diabetic normoglycemia (NDN) but also diabetic normoglycemia (DN), whose diabetes is under control. Previous studies reported that the mortality rate of patients with SIH is over twofold higher than those patients with DH [22]. Therefore, SIH, not DH, was associated with higher mortality in trauma patients [22]. However, little is known whether these trauma patients with SIH represent a distinct group with differential outcomes than those with DH. The impacts of SIH and DH on patients with traumatic femoral fractures remain unknown. Therefore, this study aimed to assess the effects of SIH and DH on the outcomes of patients with traumatic femoral fractures in comparison with those who had DN and NDN. The primary study hypothesis stated that patients with SIH had a similar worse outcome to those with DH. The mortality is measured as the primary outcome.

## 2. Methods

### 2.1. Ethics Statement

After obtaining approval (approval number: 201701325B0) from the institutional review board (IRB) of Chang Gung Memorial Hospital, a level I regional trauma center in southern Taiwan [23,24], we retrospectively selected the patients with femoral fractures and admitted via the emergency department from 1 January 2009 to 31 December 2016. According to the IRB regulations, the informed consent was waived.

### 2.2. Study Population

**Figure 1:**
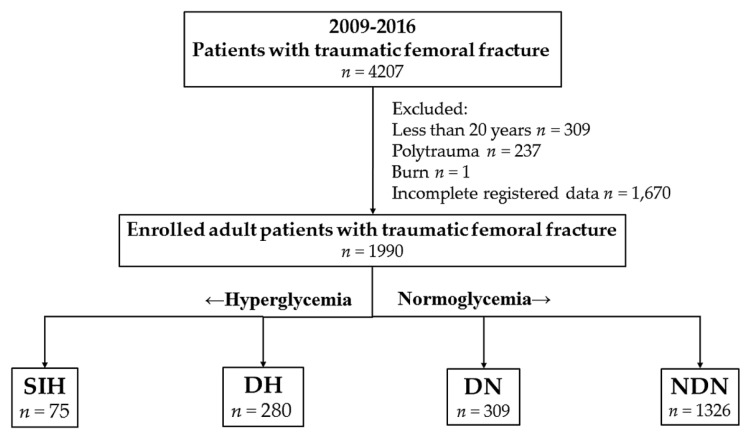
Flow chart of allocating patients with traumatic femoral fractures into four groups: stress-induced hyperglycemia (SIH), diabetic hyperglycemia (DH), diabetic normoglycemia (DN), and non-diabetic normoglycemia (NDN).

Admission hyperglycemia was diagnosed as a serum glucose ≥200 mg/dL upon arrival at the emergency department. DM was defined by patient history and/or admission HbA1c ≥ 6.5%, according to the current recommendations from the American Diabetes Association [25]. This inclusion group consisted of 4207 patients who had sustained a traumatic femoral fracture and admitted for treatment via the emergency department (Figure 1). In this study, only adult patients ≥20 years old and those who had either a history of DM or an HbA1c level upon arrival at the emergency department were included, considering that nearly 1 in 4 patients had occult diabetes based on HbA1c testing [22]. To avoid the confounding effects of injuries to other body regions in assessing patient’s mortality, the polytrauma patients who had additional AIS scores ≥3 points in other regions of the body were excluded from the study [26]. Those who had sustained a burn injury and had no available serum glucose measurement upon arrival at the emergency department were excluded from the study. In this study, the enrolled patients were allocated into four exclusive groups: (1) SIH, a serum glucose ≥200 mg/dL in the patients without DM; (2) DH, a serum glucose ≥200 mg/dL in patients with DM; (3) DN, a serum glucose <200 mg/dL in the patients with DM; and (4) NDN, a serum glucose <200 mg/dL in patients without DM. 

### 2.3. Study Variables

The following patient information were extracted from the Trauma Registry System: sex, age, sites of femoral fractures, co-morbidities, such as hypertension (HTN), coronary artery disease (CAD), congestive heart failure, cerebral vascular accident (CVA), and end-stage renal disease; serum glucose level at the emergency department; HbA1c level; Glasgow Coma Scale (GCS); Injury Severity Score (ISS), expressed as the median and interquartile range (IQR, Q1–Q3); hospital length of stay (LOS); intensive care unit (ICU) admission rates; and in-hospital mortality.

### 2.4. Statistical Analysis

The statistical analysis was done using the IBM SPSS Statistics for Windows, version 20.0 (IBM Corp., Armonk, NY, USA). Two-sided Fisher exact or Pearson chi-square tests were used to compare categorical data. Odds ratios (ORs) with 95% confidence intervals (CIs) of the associated patient conditions were presented. The primary outcome of the study was in-hospital mortality. In addition, the adjusted odds ratios (AORs) and 95% CIs for mortality were estimated through a stepwise model selection of a multivariable regression model adjusted by controlling the cofounding variables such as age, sex, co-morbidities, and ISS, with the 95% CI of this AOR calculated. Levene’s test was used to estimate the homogeneity of variance of continuous data, then one-way analysis of variance (ANOVA) with Games-Howell post-hoc test were performed to assess the differences continuous variables of groups of patients. Kruskal-Wallis nonparametric test was used to estimate whether the ISS of subgroup samples were originated from the same distribution. The continuous data were presented as mean ± standard deviation. *p*-values < 0.05 were defined as statistically significant.

## 3. Results

### 3.1. Characteristics of Patients with Femoral Fracture with SIH

A total of 1990 adult patients with femoral fracture were included in this study (Figure 1). These patients were allocated into four groups: SIH (*n* = 75), DH (*n* = 280), DN (*n* = 309), and NDN (*n* = 1326). The injury characteristics and outcomes of investigated groups of patients are shown in Table 1. As compared with NDN, SIH involved older ages but no significant differences in sex and co-morbidities were found (Table 2). Although most of the patients had a GCS of 9–12 and the rest had ≥13, the GCS score between SIH and NDN patients is not significantly different (Table 3). In addition, although only a few patients with SIH had an ISS < 16 and more had 16–24 as compared with those who had NDN, the ISS between SIH and NDN patients had no significant difference (Table 3). In relation to the patient outcomes, the proportion between SIH and NDN patients admitted at the ICU had no significant difference. Patients with SIH did not have a significant difference of hospital LOS (12.7 vs. 9.7 days; *p* = 0.104) but presented a 13.8-fold higher mortality odds ratio than those with NDN (95% CI 3.03–62.69; *p* = 0.004). Despite considering the effects of confounding variables such as age, sex, co-morbidities, and ISS, patients with SIH still had a 9.8-fold higher mortality adjusted odds than those with NDN (95% CI 1.54–62.05; *p* = 0.016).

As compared with DN, SIH had a male predominance and lesser proportion of patients with a pre-existing HTN. There was no significant difference in age and other co-morbidities. Although only a few patients had a GCS score ≥13, the GCS score between the SIH and DN had no significant difference. In addition, the SIH patients did not have a significant difference of ISS than the DN patients (*p* = 0.053). In relation to patient outcomes, the SIH group did not have a significant difference of hospital LOS (12.7 vs. 9.9 days, respectively; *p* = 0.194) than the DN group; however, the proportion of patients admitted at the ICU and the mortality odds ratio between the SIH and DN groups had no difference. Furthermore, the adjusted mortality odds ratio between the SIH and DN groups had no significant difference (AOR = 0.3; 95% CI 0.03–2.99; *p* = 0.302).

### 3.2. Characteristics and Outcomes of Patients with DH

As compared with NDN, DH had a female predominance, older age, and higher proportion of patients with pre-existing HTN, CAD, and CVA. The GCS score between the DH and NDN groups had no significant difference, regardless of the patient subgroups established based on the GCS score (≤8, 9–12, and ≥13). In addition, DH had a higher ISS than NDN. As regards the patient outcomes, the hospital LOS and the proportion of patients admitted at the ICU between the DH and NDN group had no difference. However, DH had a 6.0-fold higher mortality odds ratio (95% CI 1.60–22.52; *p* = 0.011) and a 5.8-fold higher adjusted mortality odds ratio (95% CI 1.46–22.67; *p* = 0.012) than NDN.

As compared with DN, DH did not present a significant difference in age but had less proportion of patients with pre-existing HTN. No significant differences in sex and other co-morbidities were found between DH and DN. No significant difference in GCS score and ISS were found between DH and DN, regardless of patient subgroups established based on GCS score (≤8, 9–12, and ≥13) or ISS (<16, 16–24, and ≥25). No significant outcomes in the hospital LOS, the proportion of patients admitted at the ICU, mortality odds ratio, and adjusted mortality odds ratio were found between the DH and DN groups.

## 4. Discussion

This study demonstrated that SIH and DH patients with traumatic femoral fractures presented with a higher mortality rate than the NDN patients, but not the DN patients, with or without adjustment of the differences in age, sex, co-morbidities, and injury severity. As compared with the NDN group, a 9.8-fold and 5.8-fold increase in the adjusted mortality odds ratio of SIH and DH patients, respectively, were found in this study. This result was in agreement with the report by Leto et al., who reported that an admission blood glucose level greater than 140 mg/dL was associated with higher in-hospital mortality in the patients with hip fracture [8], albeit the definition of the level of hyperglycemia is different. However, this result was, partly, not in accordance with that reported by Kerby et al., who demonstrated that, in general trauma population, the adjusted mortality was significant in patients with SIH but not significant in patients with DH as compared with the normoglycemic counterpart patients [22].

A large cross-sectional study revealed that diabetes was associated with significantly lower bone mineral density at the femoral neck and total hip [27]. Insulin resistance in type 2 DM negatively influences bone remodeling, leads to reduced bone strength of the lower femoral neck [28], and is associated with increased fracture risk [29]. Furthermore, patients with medium and high risk of osteoporosis had significantly higher odds ratio of sustaining injury in a fall accident than low-risk patients [30]. A considerable overlap in DM and osteoporosis in older adults was found due to the high prevalence of each condition [29,31]. Therefore, under the same stress and strain force, the femur bone of patients with DM is more fragile and prone to fractures than those without diabetes. Notably, in this study, the SIH group did not have a higher ISS than the NDN and DN group. In addition, the calculation of adjusted mortality was based on the different levels of injury severity. This result, in relation to admission hyperglycemia and mortality, suggested that the characteristics, not the injury severity of these patients with traumatic femoral fractures, contributed to the higher mortality of patients with admission hyperglycemia.

The pathophysiological effect associated with SIH differs from that of the DH. SIH is an acute process initiated by stress hormone with subsequent release of inflammatory cytokines in response to stress; in contrast, DH is a chronic process associated with prolonged hyperglycemia and subsequent microvascular changes [18]. Patients with diabetes are commonly at higher risk of cerebrovascular accidents, peripheral vascular disease, and renal failure [32,33,34,35,36]. In this study, SIH and DH patients did not present with a higher mortality rate as compared with DN patients. Such results indicate that the associated co-morbidity of diabetes, even if the blood sugar level in the in DN group remained normal, still accounted for a higher mortality. Some studies had revealed that the implementation of a stricter glucose control regimen in trauma patients did not lower the mortality of the patients with DH [37,38]. The prospective, randomized controlled NICE-SUGAR study was performed to delineate the optimal target range for blood glucose level in critically ill patients and found the mortality was even higher in the critically ill patients than those under conventional glucose control [39]. However, more evidences are required to support such hypothesis.

This study had several limitations. First, an inherent selection bias and unrecognized confounding factors should be mentioned in the retrospective design of the study; Second, the patients declared dead at the scene of an accident or upon hospital arrival were not included in the database, and this may have led to a selection bias, considering that the number of fatal patients is few and is prone to have a selection bias during the mortality assessment; Third, as no fix protocol was provided in dealing with the admission hyperglycemia, we could only rely on the assumption of performing a uniform management of these patients. Although the use of glycemic control in patients with SIH or DH has been inconclusive as to whether tight glycemic control reduced the morbidity and mortality, however, this unknown condition of insulin administration in patients with hyperglycemia may cause a bias in the outcome assessment.

## 5. Conclusions

This study demonstrated that SIH and DH patients with traumatic femoral fractures had higher mortality when compared with NDN patients, but not when compared with DN patients, with or without adjustment of the differences in patient’s age, sex, co-morbidities, and injury severity.

## Figures and Tables

**Table 1:** Characteristics and outcomes of the patient groups.

Variables	SIH, *n* = 75	DH, *n* = 280	DN, *n* = 309	NDN, *n* = 1326
Sex				
Female, *n* (%)	42 (56.0)	190 (67.9)	212 (68.6)	746 (56.3)
Male, *n* (%)	33 (44.0)	90 (32.1)	97 (31.4)	580 (43.7)
Age (years)	72.6 ± 13.7	72.4 ± 10.2	74.2 ± 10.2	67.1 ± 20.1
Comorbidity				
HTN, *n* (%)	37 (49.3)	186 (66.4)	235 (76.1)	544 (41.0)
CAD, *n* (%)	4 (5.3)	31 (11.1)	32 (10.4)	89 (6.7)
CHF, *n* (%)	0 (0.0)	8 (2.9)	10 (3.2)	28 (2.1)
CVA, *n* (%)	7 (9.3)	47 (16.8)	57 (18.4)	120 (9.0)
ESRD, *n* (%)	0 (0.0)	1 (0.4)	0 (0.0)	2 (0.2)
GCS	14.4 ± 1.9	14.8 ± 0.8	14.8 ± 0.9	14.8 ± 0.9
≤8	1 (1.3)	0 (0.0)	1 (0.3)	7 (0.5)
9–12	5 (6.7)	6 (2.1)	6 (1.9)	28 (2.1)
≥13	69 (92.0)	274 (97.9)	302 (97.7)	1291 (97.4)
ISS, median (IQR)	9 (9–9)	9 (9–9)	9 (9–9)	9 (9–9)
<16	72 (96.0)	280 (100.0)	309 (100.0)	1314 (99.1)
16–24	3 (4.0)	0 (0.0)	0 (0.0)	12 (0.9)
≥25	0 (0.0)	0 (0.0)	0 (0.0)	0 (0.0)
Hospital LOS (days)	12.7 ± 11.1	10.3 ± 9.0	9.9 ± 8.1	9.7 ± 7.1
ICU admission, *n* (%)	9 (12.0)	24 (8.6)	19 (6.1)	102 (7.7)
Mortality, *n* (%)	3 (4.0)	5 (1.8)	9 (2.9)	4 (0.3)

CAD = coronary artery disease; CHF = congestive heart Failure; CI = confidence interval; CVA = cerebral vascular accident; DN = diabetic normoglycemia; DH = diabetic hyperglycemia; ESRD = end-stage renal disease; GCS = Glasgow coma scale; HTN = hypertension; ICU = intensive care unit; IQR = interquartile range; ISS = injury severity score; LOS = length of stay; NDN = non-diabetic normoglycemia; SIH = stress-induced hyperglycemia.

**Table 2:** Comparison of characteristics and outcomes of categorical variables between patient groups.

Variables	SIH vs. NDN		SIH vs. DN		DH vs. NDN		DH vs. DN	
	Odds Ratio (95% CI)	*p*	Odds Ratio (95% CI)	*p*	Odds Ratio (95% CI)	*p*	Odds Ratio (95% CI)	*p*
Characteristic								
Sex		0.965		0.038		<0.001		0.845
Female, *n* (%)	1.0 (0.62–1.58)		0.6 (0.35–0.98)		1.6 (1.25–2.16)		1.0 (0.68–1.37)	
Male, *n* (%)	1.0 (0.63–1.62)		1.7 (1.03–2.88)		0.6 (0.46–0.80)		1.0 (0.73–1.47)	
Comorbidity								
HTN, *n* (%)	1.4 (0.88–2.23)	0.155	0.3 (0.18–0.52)	<0.001	2.8 (2.17–3.73)	<0.001	0.6 (0.44–0.89)	0.010
CAD, *n* (%)	0.8 (0.28–2.19)	0.813	0.5 (0.17–1.42)	0.181	1.7 (1.13–2.66)	0.012	1.1 (0.64–1.82)	0.779
CHF, n (%)	-	0.397	-	0.221	1.4 (0.62–3.02)	0.444	0.9 (0.34–2.26)	0.790
CVA, *n* (%)	1.0 (0.47–2.30)	0.934	0.5 (0.20–1.04)	0.057	2.0 (1.41–2.92)	<0.001	0.9 (0.58–1.36)	0.598
ESRD, *n* (%)	―	1.000	-	-	2.4 (0.21–26.26)	0.437	-	0.475
GCS								
≤8	2.5 (0.31–20.97)	0.357	4.2 (0.26–67.32)	0.353	-	0.613	-	1.000
9–12	3.3 (1.24–8.84)	0.028	3.6 (1.07–12.16)	0.044	1.0 (0.42–2.48)	0.974	1.1 (0.35–3.47)	0.863
≥13	0.3 (0.13–0.77)	0.019	0.3 (0.09–0.82)	0.025	1.2 (0.52–2.97)	0.632	1.1 (0.35–3.19)	0.919
ISS								
<16	0.2 (0.06–0.79)	0.042	-	0.007	-	0.241	-	-
16–24	4.6 (1.26–16.53)	0.042	-	0.007	-	0.241	-	-
≥25	-	-	-	-	-	-	-	-
Outcomes								
ICU admission, *n* (%)	1.6 (0.79–3.38)	0.179	2.1 (0.90–4.81)	0.080	1.1 (0.71–1.79)	0.619	1.4 (0.77–2.67)	0.259
Mortality, *n* (%)	13.8 (3.03–62.69)	0.004	1.4 (0.37–5.26)	0.710	6.0 (1.60–22.52)	0.011	6.0 (0.20–1.83)	0.370
Adjusted mortality, *n* (%)	9.8 (1.54–62.05)	0.016	0.3 (0.03–2.99)	0.302	5.8 (1.46–22.67)	0.012	0.6 (0.20–1.89)	0.394

CAD = coronary artery disease; CHF = congestive heart Failure; CI = confidence interval; CVA = cerebral vascular accident; DN = diabetic normoglycemia; DH = diabetic hyperglycemia; ESRD = end-stage renal disease; GCS = Glasgow coma scale; HTN = hypertension; ICU = intensive care unit; IQR = interquartile range; ISS = injury severity score; LOS = length of stay; NDN = non-diabetic normoglycemia; SIH = stress-induced hyperglycemia.

**Table 3:** Comparison of characteristics and outcomes of continuous variables between patient groups.

	**SIH, *n* = 75**	**DH, *n* = 280**	**DN, *n* = 309**	**NDN, *n* = 1326**	**Levene’s Test *p***	**F**	***p***			**Median Difference**	**Post-Hoc *p***
Age	72.6 ± 13.7	72.4 ± 10.2	74.2 ± 10.2	67.1 ± 20.1	<0.001	18.6	<0.001	NDN	SIH	−5.5	0.008
(years)									DH	−5.3	<0.001
								DN	SIH	1.6	0.774
									DH	1.8	0.142
GCS	14.4 ± 1.9	14.8 ± 0.8	14.8 ± 0.9	14.8 ± 0.9	<0.001	4.8	0.002	NDN	SIH	0.4	0.238
									DH	−0.0	0.967
								DN	SIH	0.4	0.236
									DH	−0.0	0.996
LOS	12.7 ± 11.1	10.3 ± 9.0	9.9 ± 8.1	9.7 ± 7.1	<0.001	3.9	0.009	NDN	SIH	−3.0	0.104
(days)									DH	−0.6	0.675
								DN	SIH	−2.7	0.194
									DH	−0.4	0.953
	**SIH, *n* = 75**	**DH, *n* = 280**	**DN, *n* = 309**	**NDN, *n* = 1326**	**Levene’s Test *p***	**F**	**Kruskal-Wallis *p***			**Median Difference**	**Post-Hoc *p***
ISS,	9 (9–9)	9 (9–9)	9 (9–9)	9 (9–9)	N/A	N/A	<0.001	NDN	SIH	0	0.854
Median (IQR)									DH	0	<0.001
								DN	SIH	0	0.053
									DH	0	0.630

DN = diabetic normoglycemia; DH = diabetic hyperglycemia; GCS = Glasgow coma scale; ISS = injury severity score; LOS = length of stay; N/A = not available; NDN= non-diabetic normoglycemia; SIH = stress-induced hyperglycemia.
